# Physical activity and school adaptation among Chinese junior high school students: chain mediation of resilience and coping styles

**DOI:** 10.3389/fpsyg.2024.1376233

**Published:** 2024-04-26

**Authors:** Xinbo Wu, Junwen Liang, Jiaxi Chen, Weixin Dong, Chunxia Lu

**Affiliations:** Department of Sport Education, Hunan Normal University, Changsha, Hunan, China

**Keywords:** junior high school students, physical activity, school adaptation, resilience, coping styles

## Abstract

**Background:**

As a crucial juncture in students’ educational journey, junior high school presents challenges that profoundly influence well-being and academic performance. Physical activity emerges as a pivotal factor shaping the holistic development of junior high school students. Beyond its recognized impact on physical and mental health, engaging in regular physical activity proves effective in augmenting students’ adaptability to school life. Despite its importance, the mechanisms through which physical activity influences school adaptation in junior high school students remain understudied in academic research.

**Objective:**

In exploring the potential mechanisms, this study aims to validate the mediating roles of resilience and coping styles by examining the association between physical activity and school adaptation among junior high school students.

**Methods:**

This study employed cross-sectional survey approach among junior high school students in China. Through the convenience sampling, 1,488 participants aged from 12 to 16 years old (Average age = 13.59, SD = 1.017) from two Junior high schools in Changsha City, Hunan Province were recruited to complete the Physical Activity Scale, School Adaptation Questionnaire for Junior High School Students, Resilience Scale for Adolescents, and Simple Coping Styles Questionnaire. For data analysis, the SPSS 26.0 and Amos 26.0 were used for statistical processing.

**Results:**

The results showed that physical activity exhibited a significant correlation with school adaptation (*r* = 0.656, *p* < 0.001). Resilience, positive coping style and negative coping style played partial mediating roles between physical activity and school adaptation, with the effect size were 0.229, 0.170, 0.171. The chain mediation effect size of resilience and positive coping style was 0.042, while the chain mediation effect size of resilience and negative coping style was 0.050.

**Conclusion:**

Physical activity positively predicts Chinese junior high school students’ school adaptation through resilience and coping styles, suggesting that junior high school students should engage in regular physical activity, so as to improve their resilience and positive coping styles, mitigating negative coping styles, thus promoting their school adaptation.

## Introduction

School adaptation (SA) is defined as students’ adaptation and adjustment to various aspects of school life, encompassing academic performance, interpersonal relationships, extracurricular activities, and attitudes toward school (Tomás et al., 2020). If a student is able to comprehensively achieve educational objectives, successfully complete academic pursuits, and acquire effective communication skills within the school environment, while fostering a positive outlook and values, and developing a healthy personality, then it can be determined that the student has attained a high level of school adaptation (Chen, 2003). Researches showed that successful school adaptation positively enhance students’ academic achievement (Zysberg and Schwabsky, 2021), interpersonal skills (Kiuru et al., 2020), life perspectives (Zhou, 2012), and positive character traits (Nikooyeh et al., 2017). Therefore, for junior high school students, adapting to school life is a crucial early developmental task (Li et al., 2023), an essential component of their social adaptability (Hou et al., 2022), and a significant indicator of their overall health and development level. However, it’s crucial to acknowledge that puberty, along with significant physiology (Henriques-Neto et al., 2023), psychological (Vijayakumar et al., 2023), and socio-emotional changes (Ding et al., 2023), brings various pressures impacting academic performance and interpersonal relationships (Tan et al., 2022). Concurrently, increased academic competition and a heavy workload add to the burden (Laube and Fuhrmann, 2020). Inability to effectively adapt to these modifications can potentially lead to maladaptation within the educational setting. Statistics indicate that approximately 18 to 35% of Chinese adolescents experience maladaptation issues in school (Wang, 2023). These issues not only significantly hinder students’ academic progress but may also lead to the development of psychological problems (Chen et al., 2023) such as depression and anxiety, as well as behavioral issues (Wu et al., 2023) like aggression and misconduct. Consequently, these challenges pose a potential threat to the growth of students and the stability of society. As a result, supporting students in effectively adapting to the school environment and managing challenges is of utmost importance during this critical stage.

Previous studies underscore the vital role of physical activity (PA) in fostering students’ overall well-being. Engaging in physical activity fortifies resilience (Zach et al., 2021; Guo and Liang, 2023), enhances concentration (Ou et al., 2023) and promotes positive coping strategies (Wu, 2012) and academic performance (Zhu et al., 2021). Physical activity also proves cost-efficient in enhancing school adaptation, presenting a more economical alternative than expensive academic interventions (Gao et al., 2023). In addition to physical activity, previous researches highlight the critical roles of resilience and coping styles in shaping students’ adaptation to school settings. Nevertheless, a systematic exploration of the correlation among these four elements has been lacking, limiting our comprehensive understanding of their relationship. This study marks the inaugural cross-sectional analysis examining the connection between physical activity and school adaptation, grounded in the resilience theory and social cognitive theory.

Resilience theory asserts that resilience is an ongoing dynamic process (Rutter, 2012), where individuals consistently improve their resilience through continuous learning, experiences, and personal growth (Yeager and Dweck, 2012). Emphasizing the cultivation of positive coping mechanisms within this framework is crucial for effectively managing stress and challenges (Leipold and Greve, 2009). On the other hand, reciprocal determinism is a fundamental concept within Social cognitive theory (SCT) proposed by Bandura (1977), which emphasizes the bidirectional influence among personal factors, environmental influences, and behavior. In other words, individuals do not passively react to their environment, nor are they solely influenced by their internal cognitive processes. Instead, behavior, personal factors, and environmental factors continually interact and influence each other in a dynamic manner. Within this theory, the physical activity and coping styles could be seen as the individual behaviour, and the school environment could be seen as the environmental influences. Resilience may be operationally defined as strength awareness itself—that is, the belief that one can persevere or accomplish goal-relevant tasks across varied challenges and adverse situations. In this definition, psychological resilience would fall squarely within SCT’s personal attributes (Lightsey, 2006). Building upon these theoretical foundations, this study establishes chain-mediated research models to explore the correlation between physical activity and school adaptation.

### Physical activity and school adaptation

Engaging in physical activity has emerged as a behavior conferring numerous advantages for individuals and society, including physical health, psychological well-being, and social aspects. Primarily, physical activity enhances blood flow and oxygen delivery to the brain (Nay et al., 2021), thereby augmenting students’ concentration in academic settings (Haverkamp et al., 2020). Additionally, physical activities possess the capacity to alleviate stress, anxiety, and depression (Singh et al., 2023), with neurotransmitters like endorphins released during exercise contributing to mood improvement and overall well-being (Pahlavani, 2023). Moreover, certain physical activities, like team sports and group exercise classes, frequently involve social interaction. In these settings, individuals collaborate with teammates to achieve common goals, fostering opportunities for socialization. Research suggests that engaging in such activities enhances students’ interpersonal skills (Ortega-Gómez et al., 2023), cooperation, and teamwork (Opstoel et al., 2020), culminating in a positive social experience. This positive experience, characterized by a sense of belonging and acceptance within the school environment (Smith et al., 2021), contributes significantly to students’ overall well-being (Opstoel et al., 2020).

### The mediating role of resilience

Resilience encapsulates an individual’s ability to navigate challenges and overcome setbacks by adjusting their mindset when confronted with stress, adversity, or significant setbacks (Luthar et al., 2000). According to resilience theory, despite the negative correlation between adolescent mental health and various stressors, there exists individuals with well-developed psychological states (Werner, 1984). Individuals facing stressful situations may not necessarily experience maladjustment; rather, they may exhibit strong resilience. Several studies have demonstrated a robust association between physical activity and resilience (Moljord et al., 2014; Belcher et al., 2021). Students who engage in elevated levels of physical activity display heightened resilience (Hu, 2019) and optimism (Kolovelonis and Goudas, 2018) in comparison to their less active counterparts. At the same time, resilience plays a key role as a protective mediator in coping with challenges, managing stress and maintaining emotional stability (Wang, 2023). It provides students with the psychological resources to cope with the complex emotional (Arslan, 2016), academic (Reeve et al., 2020) and interpersonal issues (Liu et al., 2021). These factors significantly influence adaptability to the school environment. Hence, association between resilience and school adaptation may exist.

### The mediating role of coping styles

Coping styles encompass the cognitive and behavioral strategies individuals employ when confronted with stress and frustration (Patterson and McCubbin, 1987), with a notable distinction between positive and negative coping styles. Researches have affirmed that physical activity among junior high school students associated with the positive coping styles (Cao and Zhang, 2006; Liu, 2016a). The higher the frequency of engagement in physical activity among students, the more adept they become at coping with problems rather than avoiding them when confronted with challenging situations (Wu, 2012). Moreover, coping styles represent significant variables influencing school adaptation (Zhang et al., 2021). Those accustomed to positive coping styles typically adopt a problem-solving approach rather than an emotionally oriented one (Stanisławski, 2019). They excel in cultivating a positive self-concept (Coelho et al., 2020), and this optimistic mindset proves beneficial in addressing academic (Marsh and Martin, 2011) and social (Miao et al., 2018) challenges. Conversely, junior high school students embracing negative coping styles, such as avoidance or giving up, may encounter difficulties in school environment (Sun et al., 2023).

### Chain mediation of resilience and coping styles

Resilience and coping styles are adaptive states displayed by individuals when confronted with stressors and adversities (DiCorcia and Tronick, 2011). Resilient junior high school adolescents, with their robust ability to handle personal and academic challenges, often see obstacles as opportunities for growth and adopt a versatile approach to problem-solving (Song and Li, 2020). This adaptability allows them to navigate academic (De la Fuente et al., 2017) and interpersonal (Guo et al., 2021) pressures with enhanced effectiveness. Conversely, individuals with diminished resilience tend to avoid challenges and exhibit apprehension toward unfamiliar experiences or difficult circumstances (Beasley et al., 2003). These individuals are more sensitive to failures and setbacks, making them susceptible to negative emotions (Yao et al., 2022). Additionally, they may harbor uncertainties about their capabilities and worth, experiencing discomfort in educational and social contexts (Liu et al., 2016).

**Figure 1:**
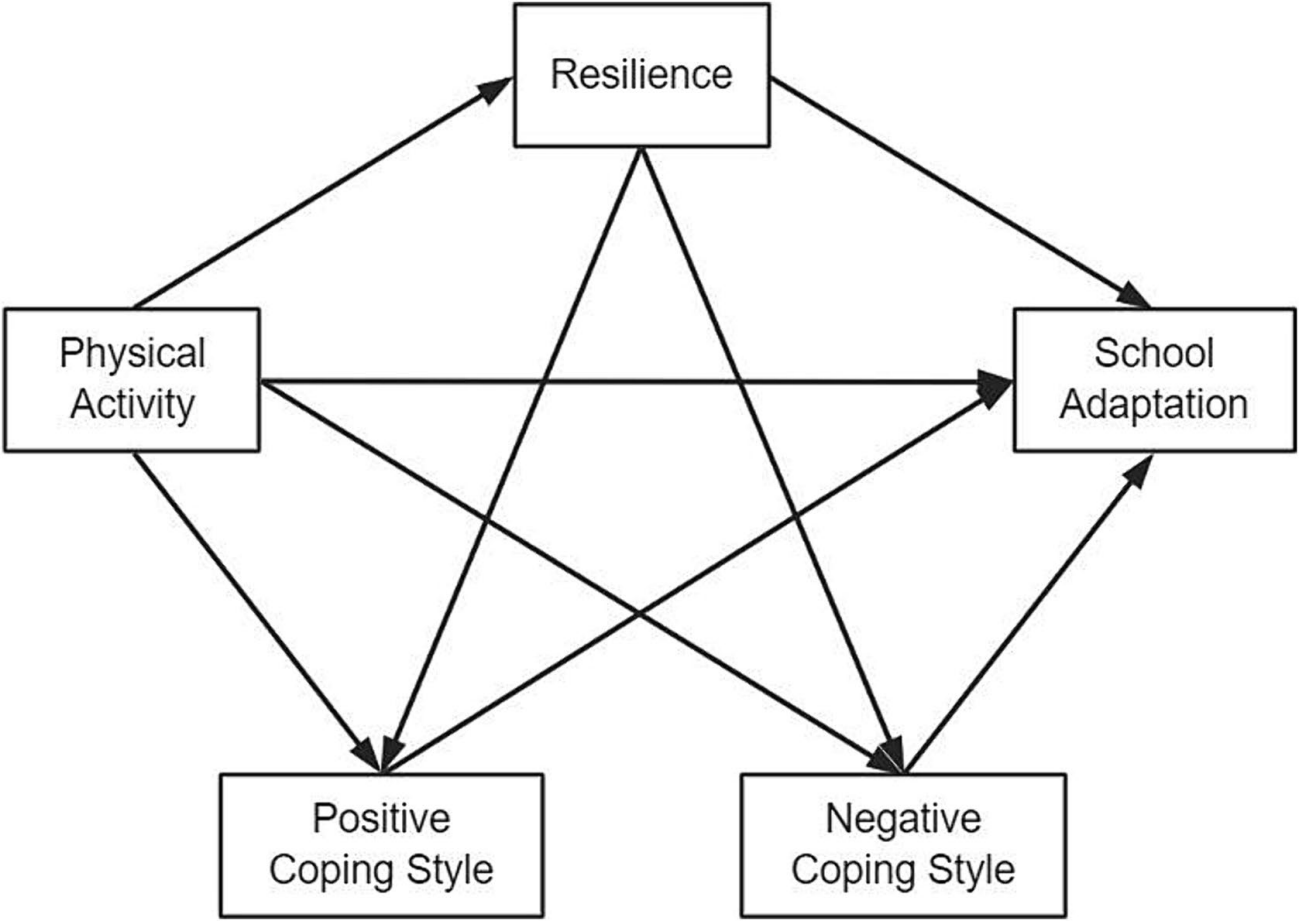
Hypothesized model of resilience and coping styles between physical activity and school adaptation,

In summary, this study aims to investigate the correlation between physical activity and school adaptation among Chinese junior high school students, and to examine whether resilience and coping styles serve as chain mediators in this relationship. The hypotheses are as followed: H1 physical activity is positively associated with school adaptation. H2 resilience mediates the relationship between physical activity and school adaptation. H3 coping styles mediate the relationship between physical activity and school adaptation. H4 resilience and coping styles play chain mediating role between physical activity and school adaptation. Figure 1 shows the hypotheses model between physical activity and school adaptation. The insights gleaned from this study contribute to a deeper understanding of the underlying mechanisms and offer a more comprehensive perspective on the factors influencing junior high school students’ school adaptation. Moreover, by providing theoretical evidence for innovative interventions and targeted support programs aimed at enhancing students’ school adaptation, this study has the potential to benefit not only junior high school students but also individuals across different age groups and educational settings.

## Method

### Research design

This study employed a cross-sectional to investigate the relationships outlined in the hypotheses. Convenience sampling was employed to select participants from March 2023 to May 2023. The survey adhered to a systematic protocol with explicit permissions secured from the school principal, class teacher, and participating students. Administered class by class during recess, participants were provided specific guidelines for self-completing the comprehensive questionnaire within a designated timeframe. Respecting students’ autonomy, the 25-min investigation allowed withdrawal at any point if desired. To ensure confidentiality and data integrity, an immediate on-site recycling process was implemented upon questionnaire completion.

### Participants and context

In this study, participants were recruited from a diverse range of schools located across Changsha city, Hunan Province, situated in the southeast region of China. To ensure a comprehensive representation, schools were carefully chosen using random sampling techniques, encompassing both urban and rural areas. Eligible schools were public or private institutions serving students in grades 9–12, with a minimum enrollment of 1,000 students, and a willingness to partake in the study. Ultimately, participants were selected using convenience sampling from two junior high schools located in Changsha. The study involved distributing questionnaires to a total of 1,630 students across 43 classes, after excluding duplicated or inconsistently answered questionnaires, a final valid sample of 1,488 questionnaires was obtained, resulting in an effective response rate of 91.2%. The participants included 769 male and 719 female students, distributed across the three school years (500 in year one, 493 in year two, and 495 in year three), with an average age of 13.59 ± 1.017 years (see Table 1). All invited participants are voluntary, thus confidentiality has been guaranteed, and written informed consent of all participants’ parents or guardians has been obtained.

**Table 1:** The demographics of the participants.

Variables	Categories	Number of participants	Percentage(%)
Gender	Male	769	51.7
	Female	719	48.3
Age	12	216	14.5
	13	509	34.2
	14	467	31.4
	15	257	17.3
	16	39	2.6
Grade	Seventh	500	33.6
	Eighth	493	33.1
	Ninth	495	33.3

### Instruments

The formal questionnaire employed in this research comprises two principal sections. The first section is dedicated to collecting essential demographic information about the respondents, encompassing variables such as gender, age, and grade level. The subsequent section incorporates well-established scales to assess various dimensions, including physical activity, resilience, coping styles, and school adaptation.

To evaluate the physical activity levels of individuals, the well-established Physical Activity Rank Scale (PARS-3) developed by Liang (1994) was utilized. This scale has been widely used among Chinese adolescents (Bai et al., 2022; Zhang and Li, 2023), particularly in cross-sectional studies. This scale encompasses three dimensions: exercise intensity (e.g., How intensely do you engage in physical activity?), exercise time, and exercise frequency. Each dimension is further stratified into five grades. Intensity and frequency of physical activity are rated on a scale from 1 to 5, while time spent on physical activity is rated on a scale from 0 to 4. The total physical activity score is computed by multiplying intensity, time, and frequency, resulting in a score ranging from 0 to 100. A higher score indicates a more substantial level of exercise engagement. In this study, the internal consistency coefficient α of the questionnaire was 0.830.

The school adaptation Scale, adapted from the revised Questionnaire by Cui (2008), has been extensively employed for assessing school adaptation among junior high school students in China (Wu et al., 2023). Comprising 27 questions (e.g., I was often distracted while studying.), the scale includes dimensions such as Routine Adjustment, Course Adjustment, Teacher-Student Relationship Adjustment, Friend Adjustment, and School Attitude Adjustment. Utilizing a five-point rating system, ranging from “1 = extremely inconsistent” to “5 = extremely consistent”, the scale employs reverse scoring for questions 3, 5, 14, 16, and 23. Here, “1 = extremely consistent” corresponds to the highest level of agreement, while “5 = extremely inconsistent” denotes the lowest level. Elevated scores in each dimension indicate a higher level of adaptability to the school environment. In this study, the internal consistency coefficient α of the questionnaire was 0.890.

The resilience Scale for Adolescents, revised by Hu and Gan (2008), comprises 27 questions (e.g., Failure always makes me feel discouraged.) distributed across five dimensions: Goal Focus, Emotional Control, Positive Cognition, Family Support, and Interpersonal Assistance. Utilizing a five-level scoring system, ranging from “1 = extremely inconsistent” to “5 = extremely consistent”, the scale employs reverse scoring for questions 1, 2, 5, 6, 9, 12, 15, 16, 17, 21, 26, and 27, with the same rating scale applied. A higher cumulative score across the selected items corresponds to an elevated level of resilience. This scale has been proven to have good validity and reliability among Chinese adolescent (Song and Li, 2020; Guo et al., 2021). In this study, the internal consistency coefficient α of the questionnaire was 0.926.

The Simple Coping Style Questionnaire, developed and consolidated by Xie (1998), consists of 20 items (e.g., When life gets tough, I can find several solutions to my problems or I relieve my worries by eating, smoking, drinking, or taking medication.) categorized into two dimensions: Positive Coping and Negative Coping. Respondents rate each item using a four-point scale, ranging from “0 = extremely inconsistent” to “3 = extremely consistent.” Questions 1 to 12 form the Positive Coping style (PCS), while questions 13 to 20 constitute the Negative Coping style (NCS). Higher scores in Positive Coping indicate a propensity to employ positive strategies in the face of challenges, whereas higher scores in Negative Coping suggest a tendency to adopt negative approaches. This scale has been proven to have good validity and reliability among Chinese students (Wu et al., 2020; Zhao et al., 2021). In this study, the internal consistency coefficient α of positive and negative dimension were 0.945 and 0.923, respectively.

### Data analysis

The research analyzed the collected data using SPSS 26.0 and Amos 26.0, with a significance level set at 0.05. To address potential common method bias, a Harman’s common method bias test was initially conducted using SPSS. Subsequently, a confirmatory factor analysis using Amos. Pearson’s correlation analysis was utilized to explore relationships among physical activity, resilience, coping styles, and school adaptation. Lastly, the study delved into examining the mediating effects of resilience and coping styles through Amos’s structural equation modeling, including an assessment of the chained mediating role of mental resilience and coping styles within this framework. The Bootstrap method was applied for evaluating the significance of the intermediate effect, with the samples being replicated 5000 times. The assessment of the mediating effect’s significance relied on whether the Bias-Corrected 95% confidence interval (CI) included zero. A non-zero inclusion within the confidence interval signifies a noteworthy mediation effect, whereas zero inclusion implies an insignificant mediation effect.

### Common method bias

The data collection method employed in this study relied on self-report measures. It is crucial to acknowledge that such methods carry the potential for common methodology bias, which could influence the research outcomes. To address this concern, Harman’s single-factor test method was utilized to examine the presence of common bias within the research data. The Kaiser–Meyer–Olkin (KMO) value, calculated at 0.956 with a significance level of *p* < 0.001, indicated the suitability of the data for exploratory factor analysis. Unrotated principal component analysis, conducted on the variable measurement questions using SPSS 26.0 software, revealed that the first principal component accounted for only 22.86% of the total variation, falling below the critical value of 40%. This suggests that common method deviation did not exert a significant impact on the study’s results.

## Results and analysis

### Correlation analysis

The present study employed Pearson correlation coefficient analysis to assess the strength and direction of the relationship between variables derived from the value of the phase relationship. The findings are presented in Table 2. Notably, the correlation coefficients between any two variables, namely PA, SA, PR, PCS, and NCS, were all statistically significant at a 1% level. Moreover, a significant positive correlation was observed between PA, PR, and PCS, and SA (*r* = 0.576, *r* = 0.499, *r* = 0.519, *p* < 0.01). Conversely, a significant negative correlation was found between NCS and SA (*r* = −0.563, *p* < 0.01), thereby providing preliminary evidence to support the plausibility of the hypothesis proposed in this study. Further investigation is warranted to validate these findings.

**Table 2:** Correlation coefficient matrix of research variables.

Variate	M	SD	PA	SA	PR	PCS	NCS
PA	30.14	24.56	1				
SA	96.94	13.84	0.576**	1			
PR	87.31	18.54	0.581**	0.499**	1		
PCS	19.41	7.45	0.475**	0.519**	0.398**	1	
NCS	8.65	6.36	−0.356**	−0.563**	−0.281**	−0.354**	1

**Indicates significance level *p* < 0.01. PA indicates physical activity, SA indicates school adaptation, PR indicates resilience, PCS indicates positive coping styles, NCS indicates negative coping styles.

### Hypothesis testing

#### Correlation between physical activity and school adaptation

By formulating a structural equation model, this study scrutinizes the correlation between physical activity and school adaptation among junior high school students. The outcomes reveal a statistically significant positive correlation between physical activity and school adaptation (*β* = 0.656, *p* < 0.001), thereby substantiating hypothesis H1.

#### The mediation effect of resilience

Based on the findings presented in Table 3, it is evident that in the analysis involving the mediating variable of resilience, the indirect effect is quantified at 0.229, 95% CI (0.190, 0.270). Moreover, the direct effect is recorded as 0.431, 95% CI (0.369, 0.485). This indicates that resilience plays a partial mediating role in the relationship. Additionally, the mediating effect contributes to 34.7% of the total effect. These findings provide substantial support for Hypothesis H2, which posits that resilience acts as a mediator between physical activity and school adaptation.

**Table 3:** Resilience bootstrap mediation effect tests.

Path	Effect size	Bias-corrected 95%CI		Effect ratio
		Lower	Upper	
Total effect	0.660	0.609	0.705	
Direct effect	0.431	0.369	0.485	68.8%
Indirect effect	0.229	0.190	0.270	34.7%

All coefficients are standardized, and the following empirical data are standardized results.

#### The mediation effect of coping styles

Moving to Table 4, the results of the test conducted with positive coping style as the mediating variable are presented. The indirect effect is determined to be 0.170, 95% CI (0.140, 0.204). The direct effect is identified as 0.489, 95% CI (0.428, 0.548). This suggests that positive coping style plays a mediating role in the relationship, and the mediating effect accounts for 25.8% of the total effect. Similarly, Table 5 displays the results of the test conducted using negative coping style as the mediating variable. The indirect effect is calculated as 0.171, 95% CI (0.146, 0.202). The direct effect is found to be 0.490, 95% CI (0.434, 0.540). This suggests that negative coping style plays a partial mediating role, and its mediating effect accounts for 25.9% of the total effect. Both positive and negative coping styles exhibit partial mediating effects between physical activity and school adaptation, supporting Hypothesis H3.

**Table 4:** Positive coping style bootstrap mediation effect tests.

Path	Effect size	Bias-corrected 95%CI		Effect ratio
		Lower	Upper	
Total effect	0.659	0.607	0.705	
Direct effect	0.489	0.428	0.548	74.2%
Indirect effect	0.170	0.140	0.204	25.8%

**Table 5:** Negative coping style bootstrap mediation effect tests.

Path	Effect size	Bias-corrected 95%CI		Effect ratio
		Lower	Upper	
Total effect	0.661	0.609	0.707	
Direct effect	0.490	0.434	0.540	74.1%
Indirect effect	0.171	0.146	0.202	25.9%

#### The mediation effect of resilience and coping styles

**Figure 2:**
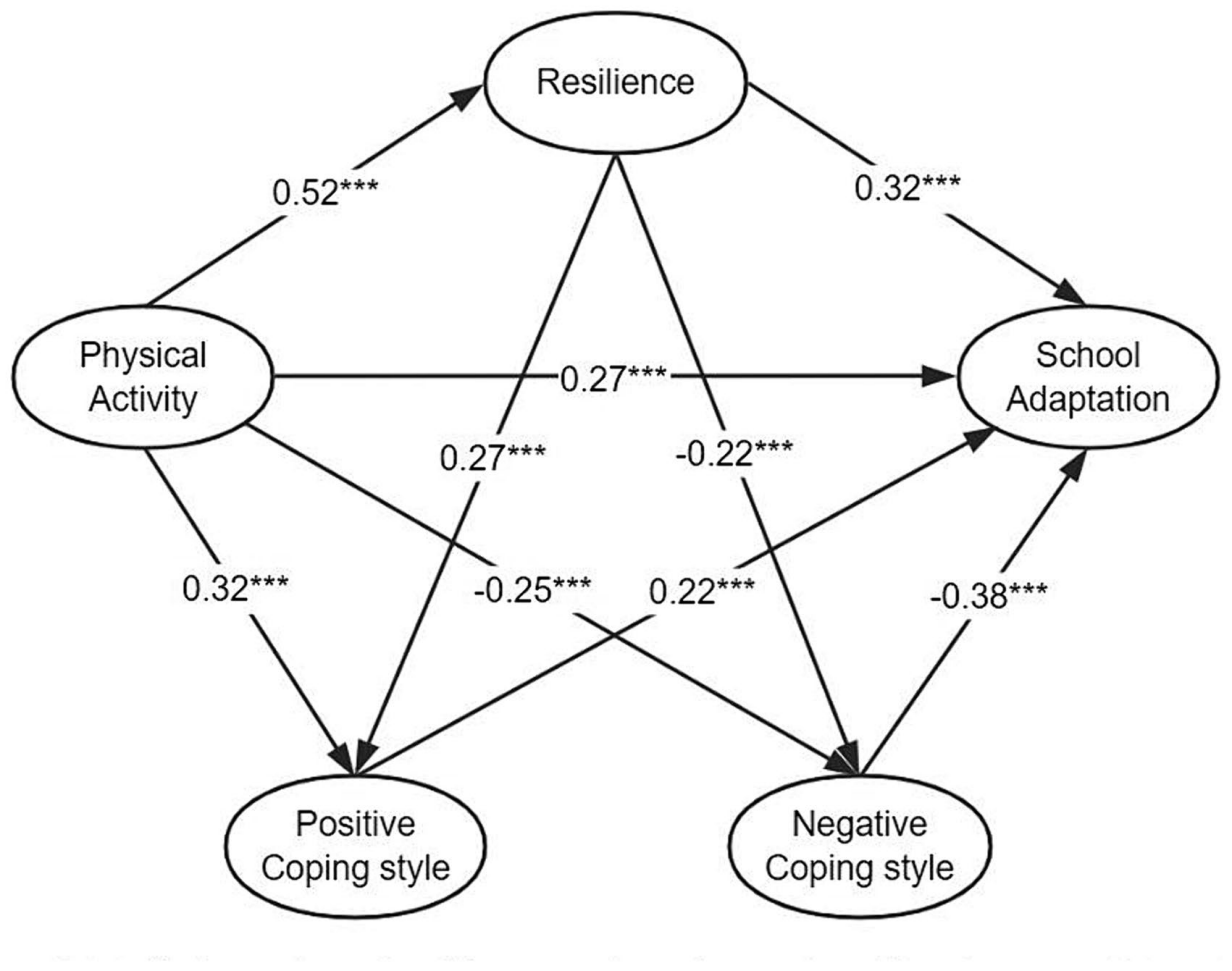
Mediating roles of resilience and coping styles effect between PA and SA. ***p < 0.001.

Following an independent examination of the mediating effects of resilience and coping style, this research proceeded to explore the interconnected mediating effects of these two variables as mediators. Consequently, a pathway model was constructed to illustrate the influence of physical activity on school adaptation, with resilience and coping style serving as mediating factors. Please refer to Figure 2 for a visual representation of the model. The hypothesis model underwent testing, yielding excellent results in terms of overall model fit: χ^2^/df = 2.623, which is less than the recommended threshold of 3. Furthermore, AGFI, GFI, NFI, IFI, TLI, and CFI all exceeded 0.9, indicating a strong fit. Additionally, the RMSEA value of 0.033 is below the recommended threshold of 0.05.

The results of the chain mediation effect test, as presented in Table 6, reveal a significant indirect effect of “physical activity → resilience → positive coping style → school adaptation” with a value of 0.030 (95% CI: 0.020, 0.043). This chain mediation effect accounts for 4.5% of the total effect. Similarly, the indirect effect of “physical activity → resilience → negative coping style → school adaptation” is also significant, with a value of 0.045 (95% CI: 0.030, 0.063). In this case, the chain mediation effect accounts for 6.6% of the total effect. Importantly, the coefficients of both pathways fall outside the range of 0 within the 95% confidence interval, indicating that resilience and coping styles play a significant role in mediating the relationship between physical activity and school adaptation, thus supporting the validity of Hypothesis H4.

**Table 6:** Test of chain mediation effect.

Path	Effect size	Bias-corrected 95%CI		Effect ratio
		Lower	Upper	
Total effect	0.680	0.627	0.725	
Direct effect	0.274	0.209	0.334	40.3%
Indirect effect	0.406	0.361	0.54	59.7%
PA → PR → SA	0.166	0.133	0.202	24.4%
PA → PCS → SA	0.069	0.049	0.093	10.1%
PA → NCS → SA	0.097	0.067	0.128	14.3%
PA → PR → PCS → SA	0.030	0.020	0.043	4.5%
PA → PR → NCS → SA	0.045	0.030	0.063	6.6%

PA indicates physical activity, SA indicates school adaptation, PR indicates resilience, PCS indicates positive coping styles, NCS indicates negative coping styles.

## Discussion

The current study explored the relationships among physical activity, resilience, positive coping styles, negative coping styles, and school adaptation among junior high school students in China. The findings illuminated that physical activity not only directly correlates the school adaptation but also exerts an indirect correlation through the pathways of resilience, positive coping styles, and negative coping styles. Five distinct mediation paths were identified: the first involving resilience as a mediating variable, the second featuring positive coping styles as the mediating variable, the third incorporating negative coping styles as the mediating variable, the fourth encompassing resilience and positive coping styles as the dual mediating variables in a chain mediation path, and the fifth involving resilience and negative coping styles as the dual mediating variables in another chain mediation path. This section outlines the major results, which are discussed alongside other relevant literature.

The findings of this research indicated the positive influence of physical activities on the school adaptation of junior high school students, which align with the research findings of Cao et al. (2018) and Gao et al. (2023). Their works indicated that the predictive role of heightened physical activity levels in junior high school students’ school adaptation, with a clear association between higher degrees of physical activity and superior levels of school adaptation. Cao also found that regular participation in physical activities enhances adolescents’ self-perception skills, facilitating the management of academic stress (Cao et al., 2018). Involvement in organized physical activity is more beneficial for adolescent’s school adaptation, due to an increase in the probability of school integration (Mosoi et al., 2020). In particular, participation in team related physical activity not only provides opportunities for forming new friendships but also eases integration into peer groups through shared interests (Zhang, 2023). Team sports cultivates teamwork spirit among students (Opstoel et al., 2020), fostering favorable peer relationships (Worley et al., 2020). Active engagement in school sports activities increases the likelihood of developing a positive attitude toward school, reinforcing a sense of school belonging and identification with campus culture (Jones et al., 2020). In summary, the involvement of junior high school students in physical activity manifests a multi-dimensional positive impact on their school adaptation, influencing not only their physical well-being but also contributing to academic, social, and emotional growth.

The findings of this research indicated that resilience serves as a crucial intermediary variable in the intricate relationship between physical activity and school adaptation. Previous research highlights the significant contribution of physical activity to students’ resilience development (Skinner and Pitzer, 2012; Mosewich et al., 2014). Low frustration experienced in different physical activity contexts (i.e., physical education and leisure-time physical activity) reports high well-being and enjoyment (Warburton et al., 2020), which would equip students with enhanced resilience. Success in physical activity also contributes to an increased sense of self-efficacy among students (Latino et al., 2023), a critical element of resilience. This heightened self-belief empowers students, reinforcing their confidence in academic pursuits (Komarraju and Nadler, 2013) and fostering a positive learning attitude and adaptability (Zimmerman, 2000), thus facilitating their adaptation to the school environment (Nowland and Qualter, 2020). Additionally, students actively participating in high-intensity physical activities demonstrate heightened perseverance and optimism compared to peers with lower activity levels (Huang, 2020), with the varying intensity of these activities further enhancing students’ mental endurance (Hu, 2019). A robust connection exists between resilience and school adaptation, where higher resilience correlates with improved emotional regulation (Huang et al., 2023) and social skills (Martinsone et al., 2022), thereby fostering overall school adaptation. Consequently, the mediating role of resilience plays a pivotal part in establishing a connection between physical activity and successful adaptation to school life.

The findings of this research indicated that coping styles act as mediator between physical activity and school adaptation among junior high school students. Put differently, an increase in the physical activity levels of these students corresponds to a likelihood of embracing positive coping styles (Chen et al., 2013; Liu, 2016b; Yang et al., 2020), thereby contributing to an enhanced school adaptation (Zhang et al., 2021). Yang Jian’s research reveals a significant positive correlation between the intensity of physical activity and its efficacy in fortifying confidence and determination among junior high school students (Yang et al., 2020). Specifically, those engaging in elevated levels of physical activity exhibit heightened resilience and unwavering personal resolve. When confronted with challenges, this demographic transitions from evading difficulties to actively seeking solutions and confronting obstacles, fostering heightened adaptability to the school environment (Bao, 2012; Shi et al., 2015). On the contrary, students who are not very physically active often resort to negative coping styles (Cheng et al., 2023), which are linked to insufficient adaptation to school (Han et al., 2016). Previous researches have also found that junior high school students with a negative attitude toward physical activities often avoid participating in such pursuits, skip regular exercise, or deviate from established training routines (Nelson et al., 2010). Students accustomed to employing negative coping styles may resort to evasion or surrender when facing life or academic challenges (Cherkil et al., 2013), leading to a decline in self-efficacy and the accumulation of negative emotions (Qu et al., 2023). This renders it more challenging for them to navigate the pressures and challenges inherent in school life and learning. Furthermore, using negative coping styles may result in conflicts and isolation with peers and teachers (Henderson et al., 2003), leading to feelings of loneliness or exclusion (Elahe et al., 2017). It would impact the relationships between students and their peers and teachers (Shen et al., 2021), leading to a decline in the level of school adaptation. In summary, it can be observed that both positive and negative coping styles play a mediating role in the connection between physical activity and school adaptation in junior high school adolescents.

The findings of this research indicate the relationship between physical activity and school adaptation, intricately mediated by resilience and coping styles among junior high school students. Specifically, active participation in sports activities significantly contributes to the cultivation of resilience (Belcher et al., 2021). Elevated resilience levels enhance the likelihood of adopting positive coping styles. These positive coping mechanisms play a pivotal role in shaping students’ adaptation within school. Conversely, students who abstain from or resist participation in physical activity correlates with diminished resilience compared to those with elevated physical activity (Li et al., 2022). Individuals with lower resilience levels exhibit inadequate coping abilities when faced with setbacks and pressures (Zou et al., 2024), such as avoidance and self-blame (Wang et al., 2020). These negative coping mechanisms impede effective problem resolution, leading to the postponement or neglect of issues and, consequently, exacerbating challenges in school adaptation (Wu, 2012). In summary, a robust correlation exists between individuals’ coping styles and their resilience. Positive coping styles and coping flexibility demonstrate a positive association with higher resilience, whereas negative coping styles exhibit a negative association with lower resilience.

### Research deficiencies and future directions

Although this study has provides valuable insights into fostering adaptive development among junior high school students within the school environment, some limitations also require special attention. Firstly, the cross-sectional design impedes the establishment of definitive causal relationships between variables without longitudinal data. Secondly, although the sample size meets statistical criteria, the limited geographical distribution coverage, confined to two middle schools in Changsha, Hunan Province, may not adequately represent the characteristics of the entire population. Lastly, due to time and energy constraints, the study primarily employs Likert scale-based questionnaires, lacking in-depth interviews with respondents.

In future studies, researchers could enhance the study’s depth by incorporating longitudinal follow-up or experimental interventions to gain a more comprehensive understanding of how physical activity influences the school adaptation of junior high school students. Future investigations should aim to broaden the sampling area, improving the generalizability of research findings and yielding more robust conclusions. Additionally, researchers can consider using a combination of quantitative and qualitative research methods to collect diverse data and materials, contributing to a richer analysis of the phenomena under investigation.

## Conclusion

This study indicated the correlation between physical activity and the school adaptation (Mosoi et al., 2020; Yan et al., 2020; Bai et al., 2022; Qin and Qin, 2023), resilience (Moljord et al., 2014; Hu, 2019; Belcher et al., 2021), coping styles (Cao and Zhang, 2006; Liu, 2016a) of junior high school students which aligns with previous researches. Furthermore, the resilience and coping styles of these students emerge as key factors impact school adaptation, each playing a distinct and partial mediating role in the relationship between physical activity and school adaptation. More interestingly, the intricate dynamics of this relationship are revealed as resilience and positive coping style collaboratively form a chain mediating role between physical activity and school adaptation, alongside resilience and negative coping styles.

Based on these findings, recommendations propose that: (i) Schools and parents should prioritize physical activity for junior high school students. Programs should offer diverse activities like team sports or individual exercises to cater to different preferences. (ii) Given the importance of resilience, efforts should be made to teach resilience-building strategies in family education, school curricula, and extracurricular activities. This can include workshops or training sessions aimed at developing resilience skills. (iii) Family, schools, and educators should focus on fostering positive coping styles among students. Support and resources should be provided to help students develop effective coping strategies, such as seeking social support and problem-solving. Interventions should address negative coping strategies through counseling or peer support programs. (iv) Recognize the connection between resilience, coping styles, and physical activity in influencing school adaptation. Future research and interventions should consider this complex interplay and involve collaborations between educators, psychologists, and health professionals to develop comprehensive strategies. By implementing these suggestions, families and educational institutions can enhance the school adaptation and overall well-being of junior high school students.

## Data availability statement

The raw data supporting the conclusions of this article will be made available by the authors, without undue reservation.

## Ethics statement

Ethical approval was not required for the study involving humans in accordance with the local legislation and institutional requirements. Written informed consent for participation in this study was provided by the participants’ legal guardians/next of kin.

## Author contributions

XW: Conceptualization, Data curation, Formal analysis, Methodology, Software, Validation, Writing – original draft, Writing – review & editing. JL: Conceptualization, Data curation, Investigation, Methodology, Project administration, Software, Validation, Writing – original draft, Writing – review & editing. JC: Data curation, Formal analysis, Validation, Software, Writing – review & editing. WD: Conceptualization, Project administration, Resources, Supervision, Visualization, Writing – review & editing. CL: Conceptualization, Project administration, Resources, Supervision, Visualization, Writing – review & editing.
